# Practical Notes

**Published:** 1877-04-20

**Authors:** 


### PRACTICAL NOTES.

GINGER BEER.– White sugar 5 lbs.; lemon juice 1 gill; honey ¼ lb.; bruised ginger 5 ounces; water 4½ gallons. Boil the ginger 30 minutes in 3 quarts of the water; when cold put in the other ingredients and strain; add the white of an egg well beaten, with a teaspoonful of lemon essence. In four days bottle.—Scientific American.

SWOLLEN JOINTS FROM RHEUMATISM.—Dissolve ¼ lb. of saltpetre in the smallest quantity of water possible and add alcohol in small quantities at a time until 1 pint is used, constantly stirring, otherwise the salt will be precipitated. Bathe the parts.

AN EXCELLENT sedative water for external application for bruises or aches is composed of ammonia 2 ozs.; tinct. camphor 2½ drachms; common salt 2 ozs., and water 2 pints. Mix and dissolve without heat.—Scientific Amer.

FOR BURNS apply cotton batting, or old, soft linnen, smeared in a mixture prepared by beating together the whites of eggs and melted lard, in the proportion of a tablespoonful lard to each egg.

FALLING HAIR.—Glycerin, and tinct. capsicum each 2 oz; oil bergomot 1 drachm; mix and perfume to suit. Dress the hair with this. Wash the head occasionally with soft water and fine soap.

LEMONS may be kept by varnishing them with a solution of shellac in alcohol.

A PINT of milk taken each night before going to bed, will fatten the leanest—it is said.

STOVE BLACKING.—Mix black lead well with white of eggs. Put on with paint brush and when dry polish with hard brush.

TOOTHACHE.—The pain is very often caused by acid saliva. When this is the case, pledgets of cotton soaked in a strong solution of bicarbonate of soda and water will give instant relief.

DENTRIFIC.—Castile soap ½ drachm; dissolved in alcohol 1 ounce; water ¾ ounce; glycerine ¼ ounce; color with cochineal and flavor with pepermint, winter green and clove oil.

LIME WATER.—Slake 4 oz. lime with a little distilled water, then add distilled water to make 1 gallon. Cover the vessel and set aside for 3 hours. Pour off the clear liquor for use.

HYDRO BROMIC ETHER, M. Rabuteau says, holds an intermediate place, as an anæsthetic, between chloroform, bromoform and ether. This is a new anæsthetic.

FOR ITCHING CHILBLAINS.—Oil turpentine 2 oz; camphor 3 drachms; oil cajeput 1 drachm. Mix and rub in with gentle friction.

MUSCULAR RHEUMATISM is said to be cured by one drop of liquor ammonia in a glass of water every two or three hours.

ONE PINT of mustard seed in a barrel of cider, after it has fermented, will keep it sweet. Bung the barrel tight.

A GILL of glue dissolved in water to which a tablespoonful of glycerine is added will stick leather, paper or wood

COLOGNE WATER, (*Nice Medical,*) has been found by Dr. Hugues to induce anæsthesia in several cases.

HARNESS BLACKING.—Dissolve 5 or 6 sticks of block sealing wax in a pint of alcohol.

FOR CRACKED SKIN OF HANDS, use spermaciti ointment.

GASOLINE forms a nice and efficacious application to extensive burns.

